# Upper Gastrointestinal Bleed in a Young Male- A Rare Presentation of Eosinophilic Gastroenteritis

**DOI:** 10.7759/cureus.7059

**Published:** 2020-02-20

**Authors:** Shivani Priyadarshni, Balarama K Surapaneni, Kairavee Dave, Steven Kaplan, Nehal Patel

**Affiliations:** 1 Internal Medicine, Aventura Hospital and Medical Center, Aventura, USA; 2 Internal Medicine/ Graduate Medical Education, Aventura Hospital and Medical Center, Aventura, USA; 3 Graduate Medical Education, Aventura Hospital and Medical Center, Aventura, USA; 4 Gastroenterology, Aventura Hospital and Medical Center, Aventura, USA; 5 Internal Medicine, Avnentura Hosptial and Medical Center, Aventura, USA

**Keywords:** eosinophilic gastroenteritis, upper gi bleeding, steroid-responsive

## Abstract

Eosinophilic gastroenteritis (EGE) is a rare idiopathic disease affecting multiple organs (stomach and small intestine) of the digestive tract. It is characterized by eosinophilic infiltration of the bowel wall to a variable depth and symptoms associated with gastrointestinal tract disease. The prevalence of this condition is ranging from 8 and 28 per 100,000. We present a rare presentation of EGE manifesting as upper GI bleeding.

A 28-year-old male with PMH of EGE, duodenal ulcers, and stricture presented to the hospital with the chief complaints of three episodes of dizziness and melena over one day. His home medications included prednisone, montelukast, and pantoprazole. On admission, he was found to be tachycardic (150) while other vital signs were stable. Physical examination revealed cold, pale and clammy skin but was otherwise normal on examination. Initial labs showed hemoglobin (hgb) of 9.3. His hospital course was complicated with 1 episode of large volume hematemesis >1.5 L and brief loss of consciousness for which a code rapid response was called. On day 2, the hgb dropped to 5.7 and the patient received a blood transfusion. Emergent endoscopy (EGD) revealed high-grade duodenal stenosis, severe pyloroduodenal deformity and a duodenal ulcer with the visible vessel. Two clips were deployed blindly. Epinephrine could not be injected due to hard and fibrotic tissue around duodenal stenosis. The Interventional Radiology team was consulted and emergent angiography was done which revealed active bleeding from a branch of the gastric artery. Embolization was done and hemostasis was achieved successfully. He needed 5 units of PRBC transfusion in total. He was treated with pantoprazole twice a day intravenously since admission. For his known duodenal stricture, the surgical team was consulted. No acute surgical intervention was recommended. On discharge, he was sent home with pantoprazole 40 mg twice a day, slow tapering of prednisone and close follow up with gastroenterology, surgery, and primary care doctor within 1 week. The purpose of this case report is to increase awareness about this clinical condition among medical professionals.

## Introduction

Eosinophilic gastroenteritis (EGE) is a rare idiopathic disease affecting multiple organs (stomach and small intestine) of the digestive tract. It is characterized by eosinophilic infiltration of the bowel wall to a variable depth and symptoms associated with the gastrointestinal tract. In some cases, inflammation may include the esophagus, the distal intestine, as well as the colon. This inflammation occurs without any other known cause of tissue eosinophilia [1,2]. Klein et al classified EGE into 3 subtypes predominant mucosal, muscular or subserosal [3]. The prevalence has been found to be ranging from 8 and 28 per 100,000 [4,5]. We report a rare presentation of Upper GI bleed in a young male with a diagnosis of EGE. EGE can affect patients of any age. However, in adults, EGE presents in the 3rd through 5th decades. The peak age of onset of EGE is in the 3rd decade. EGE can affect both genders; however, a slight female preponderance has been reported in many studies.

## Case presentation

Our patient is a 28-year-old male with a past medical history of EGE, duodenal ulcers, and stricture who presented to the hospital with chief complaints of 3 episodes of dizziness and melena for one day. The patient reported that before coming to the hospital, he was shopping at a mall, where he experienced dizziness and then had a dark black colored bowel movement. The patient recently came from Canada. He denied any other past medical history or any past surgical history. He reported allergy to peanuts. His home medications included prednisone, montelukast, and pantoprazole daily. 

**Figure 1:**
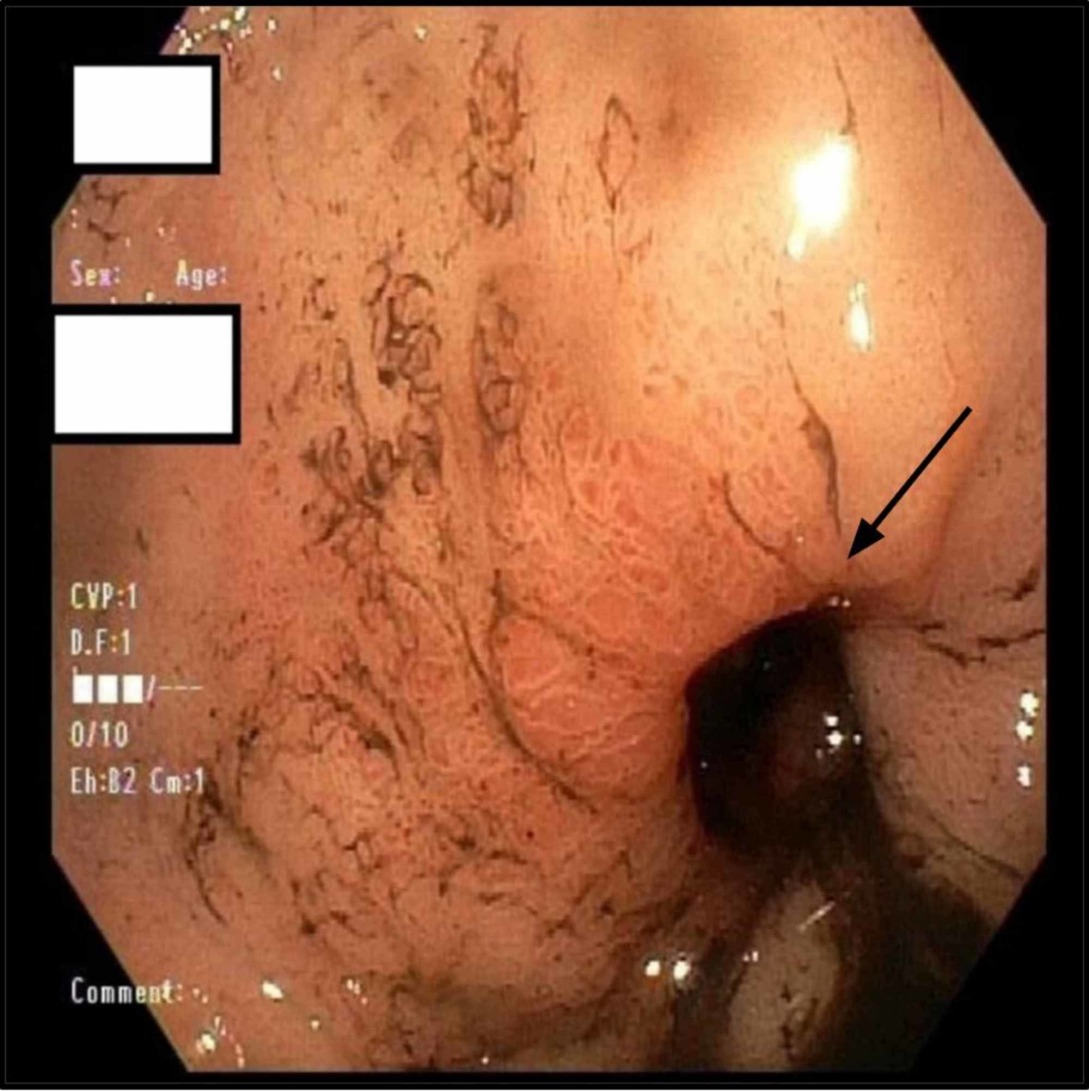
Duodenal bulb (black arrow)

**Figure 2:**
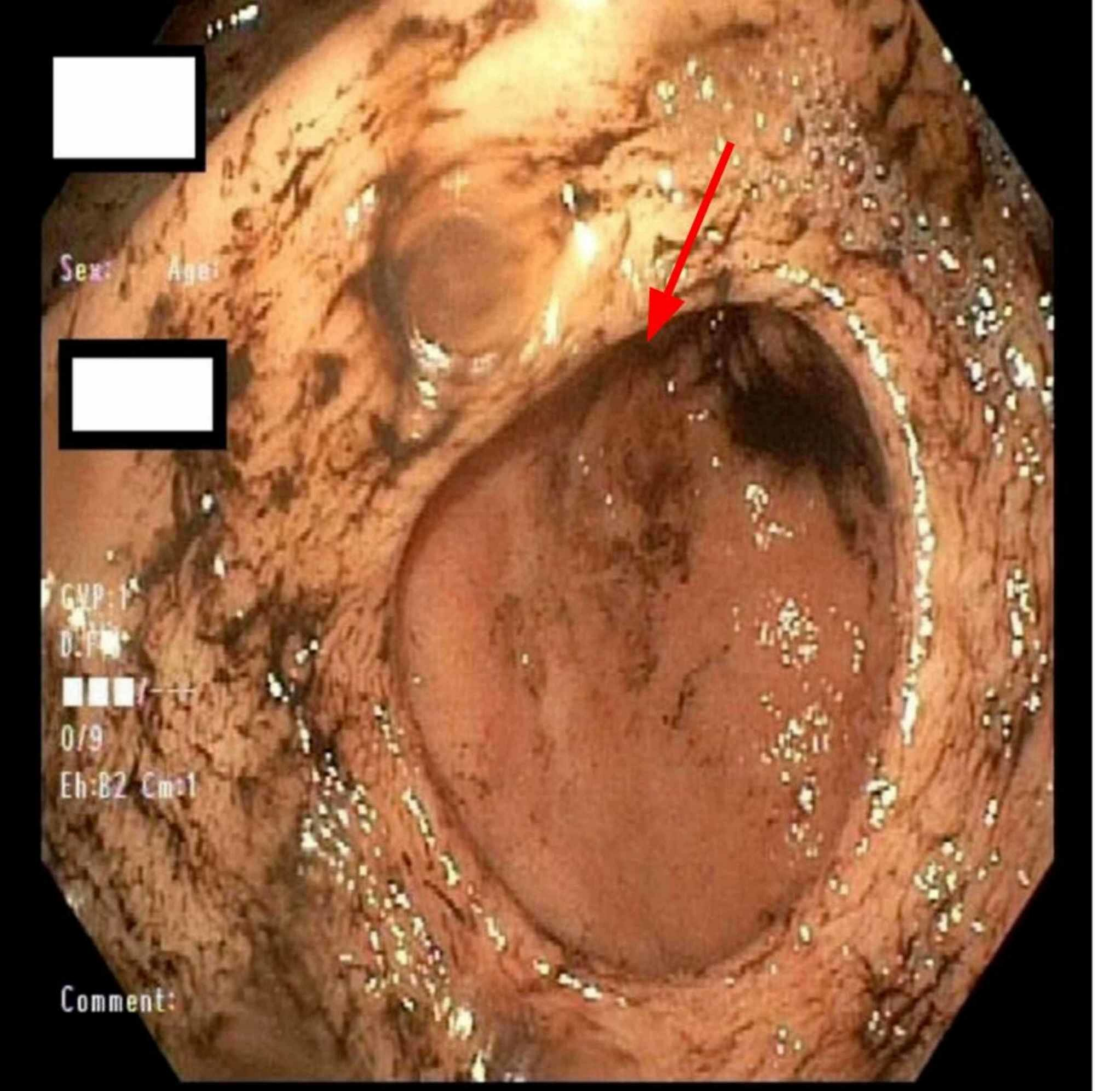
Prepyloric deformity (red arrow)

**Figure 3:**
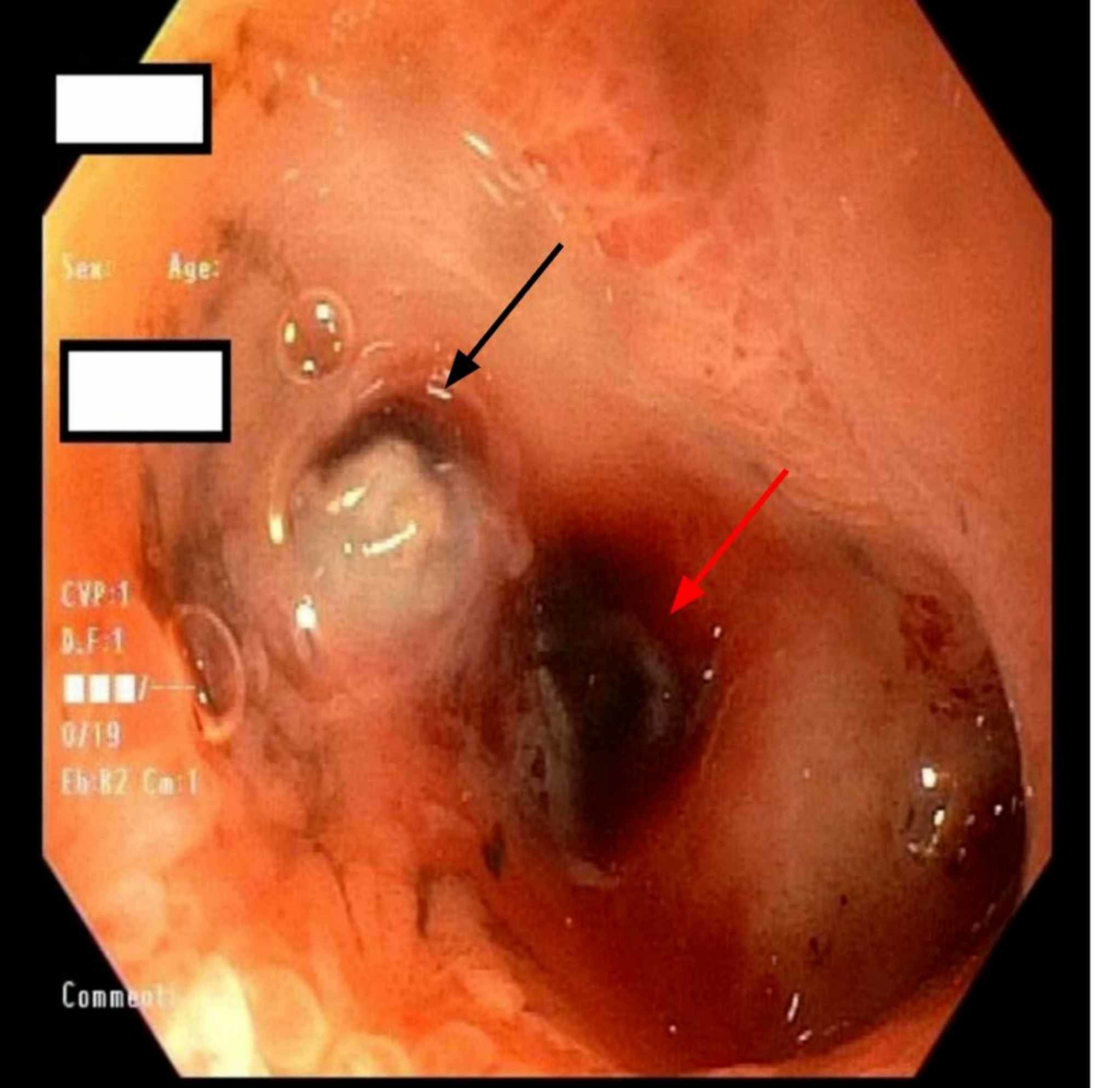
Second portion of duodenum with ulcer (black arrow) and visible vessel (red arrow)

On admission, his vitals were obtained and he was found to be tachycardic with a heart rate of 150. Initial lab workup done in the emergency department showed hemoglobin (hgb) of 9.3. Soon after admission, the patient had an episode of large volume hematemesis >1.5 L and brief loss of consciousness for which a code rapid response was called. Between days 1 and 2, his hgb dropped from 9.3 to 5.7. The Patient was initially stabilized by intravenous crystalloids and was transferred to the ICU. The patient received blood transfusions and his hgb improved significantly. The patient was managed conservatively with continuous monitoring in the ICU. Emergent endoscopy (EGD) revealed high-grade duodenal stenosis, severe pyloroduodenal deformity and a duodenal ulcer with a visible vessel. Two clips were deployed blindly during the EGD (figure 1-3).

Epinephrine could not be injected due to hard and fibrotic tissue around the duodenal stenosis. The Interventional radiology (IR) service was consulted. The patient was taken for angiography which revealed active bleeding from a branch of the gastric artery. Coiling and subsequent embolization were done by the IR service. The patient received 5 units of RBCs total and continued on pantoprazole intravenous twice daily. For his known duodenal stricture, surgery was consulted with a recommendation of no acute surgical intervention in the hospital was provided. On discharge, he was sent home with pantoprazole and prednisone and close follow up with gastroenterology, surgery and primary care doctor.

## Discussion

EGE can affect patients with any age; however, EGE is more common in the pediatric population. In adults, EGE typically presents between the 3rd and 5th decade of life with a female predominance in the USA [5]. There are various risk factors for EGE which include genetic factors observed in familial cases, higher socioeconomic status, Caucasian race and excess weight [6-8]. EGE is seen in many patients with a history of seasonal allergies, atopy, food allergies, asthma, and elevated serum IgE levels, suggesting an association with hypersensitivity in the pathophysiology of EGE [9].

The pathophysiology of EGE is not fully understood. Many pathways have been studied showing EGE could have either IgE dependent or independent pathways. It is been shown that eosinophil recruitment leads to a T-cell mediated chemokine production by eosinophils [9-11]. There are 3 clinical criteria used to diagnose EGE: 1) Presence of GI symptoms (abdominal pain, nausea, vomiting, early satiety, diarrhea, weight loss, malabsorption, failure to thrive, etc. 2) Eosinophilic infiltrates in various parts of the gastrointestinal tract and 3) Exclusion of causes of tissue eosinophilia such as drug reactions or parasitic infections. The clinical features of EGE vary depending on the location, the extent of the organ involvement, and the layers of bowel with eosinophilic infiltrations [9-11].

The manifestations on EGD vary from mild erythema and mucosal wall thickening to frank ulcerations and perforations. Initial work up to diagnose EGE includes 1. Peripheral eosinophils count which is usually elevated in EGE patients, however, it may be normal in approximately 20 % of patients 2. Complete blood count to look for iron deficiency anemia (IDA) (EGE can be associated with IDA). 3. Tests for malabsorption 4. Serum immunoglobulin E levels can be elevated, especially in the pediatric population. The confirmatory diagnosis of EGE is established by demonstrating eosinophilic infiltration on endoscopic biopsy. The endoscopic biopsy usually shows more than expected eosinophils on microscopic examination; however, there is no exact cut off for the number of eosinophils per high power field to diagnose EGE [9-11].

Initial treatment of EGE involves dietary modifications which include different types of diets such as an empiric elimination diet, a six-food elimination diet, or an elemental diet. These diets are focused on the elimination of all potential food allergens. If dietary measures do not improve symptoms, then a trial of corticosteroids is suggested. The mainstay therapy has been corticosteroids treatment as it has demonstrated efficacy in various studies [12-14]. However, there have been relapses of EGE on withdrawal of steroids, and long term use of corticosteroids has been associated with multiple adverse effects ranging from osteoporosis to a cushingoid state. For Non-IgE independent EGE which is usually related to food allergies, the use of elimination diets has been recommended [15]. The food elimination diet has been used recently in pediatric populations with EGE [16]. 

This work has been presented as an abstract to the American College of Gastroenterology (ACG) annual meeting 2019 (Shivani P, Surapaneni BK, Dave K, Patel N, Yella PR, Cantave RT, Kaplan SR. Upper GI Bleed in a Young Male: A Rare Presentation of Eosinophilic Gastroenteritis. Oct 2019. https://journals.lww.com/ajg/Fulltext/2019/10001/Upper_GI_Bleed_in_a_Young_Male__A_Rare.2035.aspx).

## Conclusions

Our patient presented as a rare presentation of upper GI bleed with a diagnosis of eosinophilic gastroenteritis (EGE). EGE is an inflammatory disorder characterized as eosinophilic infiltration of the gastrointestinal tract. EGE can affect patients of any age but typically presents in the third through the fifth decades. The pathogenesis EGE is poorly understood, but available data suggest an allergic component. Approximately half of the patients have a history of various types of allergies. Confirmatory diagnosis is made by endoscopy. The mainstay of treatment includes dietary modifications and steroids.
